# Supplementary material for: Maternal ranging strategies facilitate offspring social play at energetic cost in the most solitary ape
Source: Proc Natl Acad Sci U S A. 2026 Oct 6;123(42):e2621225123. doi: 10.1073/pnas.2621225123 (PMC13684041; doi:10.1073/pnas.2621225123)
Supplement: Supplementary file 1 [file pnas.2621225123.sapp.pdf]

Supporting Information for

Maternal ranging strategies facilitate offspring social play at energetic cost in the most solitary ape

Odd T. Jacobson, Alison M. Ashbury, Brendan J. Barrett, Margaret C. Crofoot, Paulina Kukofka, Julia A. Kunz, Sri Suci Utami Atmoko, Caroline Schuppli, Erin R Vogel, Carel P. van Schaik, and Maria A. van Noordwijk

Odd T. Jacobson.
E-mail: ojacobson@ab.mpg.de

This PDF file includes:

Supporting text
Figs. S1 to S14
Tables S1 to S11
SI References

Supporting Information Text

Materials and Methods

Estimating pairwise relatedness from matriline pedigrees. We assigned a coefficient of maternal relatedness (r) to each female-female dyad in the analysis ($n = 71$ dyads, 282 dyad-years, among 14 mothers; Figure S1) using maternal pedigrees compiled from long-term demographic records of the Tuanan Orangutan Research Project. Following standard pedigree-based coefficients (1), mother-daughter dyads were assigned $r = 0.5$ ($n = 6$); maternal half-sisters ($n = 3$) and grandmother-granddaughter dyads ($n = 2$) were assigned $r = 0.25$; aunt-niece dyads $r = 0.125$ ($n = 4$); and maternal cousins $r = 0.0625$ ($n = 1$). Females with no documented common maternal ancestor in the pedigree were assigned $r = 0$ ($n = 55$).

We restricted our relatedness measure to the maternal line. Although paternities are available for a subset of Tuanan individuals (2), what matters for the behavioral questions examined here is *knowable maternal relatedness*: the kin a female has had life-long social exposure to through her mother. Female orangutans are strongly philopatric and remain near their natal range, whereas males disperse widely and as adults range and reproduce far beyond their birthplace (3–5). A female's maternal kin therefore form the stable social environment in which she develops, while her paternal half-sibs are typically scattered across the landscape and cannot be recognized through maternal social bonds. All values correspond to demographically observed mother-offspring links in the long-term records, with no values inferred or estimated.

Movement data processing and space-use estimation. We estimated annual home ranges, pairwise home-range overlap, and daily path length (DPL) using the `ctmm` R package (6). Continuous-time movement models account for temporal autocorrelation, irregular GPS sampling, and location error, all of which are common in nest-to-nest focal follows with handheld GPS devices. We specified a user-equivalent range error (UERE) corresponding to location errors of approximately 2-29 m, consistent with handheld GPS performance under tropical forest canopy. Location data used for annual home-range estimation included both full-day and partial-day follows, whereas DPL analyses were restricted to full-day follows, defined as follows beginning at morning nest departure and ending at evening nest arrival.

Before estimating annual home ranges, we excluded mother-years with clearly inadequate sampling coverage by requiring at least 8 observation days, 3 unique weeks, and 50 GPS locations. For each retained mother-year, we fitted candidate CTMMs and selected the best-supported model using AIC_c. We then estimated annual home ranges as autocorrelated kernel density estimates (AKDEs) (7) with optimal weights to reduce bias from uneven sampling (8). We retained only home ranges with acceptable diagnostics, based on visual inspection of empirical and model-predicted variograms and an effective sample size for area ≥ 3. This yielded 99 annual home ranges from 14 mothers with sufficient annual spatial coverage.

We quantified pairwise home-range similarity as the Bhattacharyya coefficient (BC) between utilization distributions using `ctmm::overlap()`. This procedure propagates uncertainty from the underlying CTMM fits into 95% confidence intervals on BC, which we carried into downstream Bayesian models as measurement error on the response. We estimated DPL using the continuous-time speed and distance (CTSD) method (9). For each full-day follow, we fitted a per-day CTMM and multiplied the estimated mean speed by the total daily sampling duration. This approach is robust to variation in sampling rate, path tortuosity, and location error. For each day, we extracted the CTSD point estimate and its 95% confidence interval, which we propagated into downstream Bayesian models as measurement error on the response. This yielded 2210 daily path lengths from 14 mothers with eligible full-day follows. The sample sizes differed between annual home-range and daily path-length analyses because the two metrics required different forms of sampling coverage.

Environmental covariate processing. We characterized local food availability using the fruit availability index (FAI), derived from long-term phenological monitoring at Tuanan, in which trained researchers recorded the percent of marked trees bearing fruit along permanent transects (10, 11). FAI provides a direct monthly estimate of fruit abundance within the study area and was matched to each observation window by month (Figure S4).

We complemented FAI with the Enhanced Vegetation Index (EVI), a satellite-derived proxy for canopy greenness designed to remain sensitive in dense tropical canopies where NDVI often saturates (12). We extracted EVI from the MODIS MOD13Q1 Collection 6.1 product, which provides 250 m, 16-day composite imagery. Using the `MODISTools` R package (13), we retrieved all pixels within a 6×6 km window centered on Tuanan Research Station. We averaged EVI values across pixels within each 16-day composite and then across composites within each month. Because satellite vegetation indices cannot resolve specific phenophases such as fruiting (14), we interpret EVI as an index of broad-scale leaf cover and drought-driven productivity variation rather than food abundance per se (Figure S5).

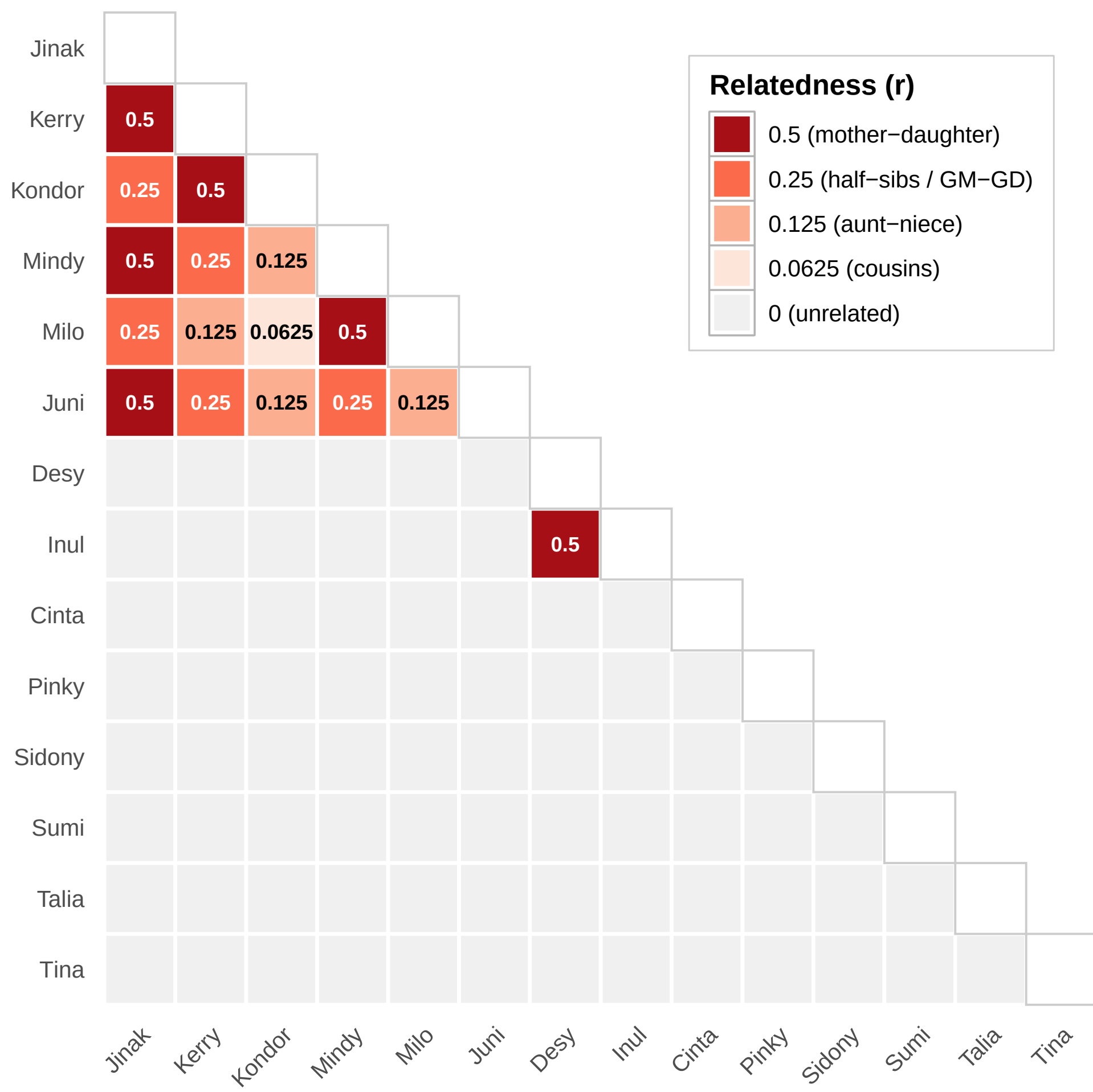

Fig. S1. Maternal pedigree relatedness (r) among the 14 mothers included in the Tuanan dyadic analyses. Each cell shows the coefficient of relatedness for one female-female dyad, derived from long-term maternal pedigrees of the Tuanan Orangutan Research Project. Cell color encodes relatedness category: $r = 0.5$ for mother-daughter; $r = 0.25$ for maternal half-sisters and grandmother-granddaughter pairs (both share, on average, a quarter of their genome through the maternal line); $r = 0.125$ for aunt-niece; $r = 0.0625$ for maternal cousins; and $r = 0$ for pairs with no documented common maternal ancestor. Cells along the diagonal are left blank because relatedness is not defined for an individual to herself. Females are ordered to keep matrilineally related individuals adjacent: the upper-left block comprises Jinak's matriline (her daughters Kerry, Mindy, Juni; her granddaughters Kondor and Milo), followed by the Desy-Inul mother-daughter pair, with the remaining six females belonging to separate matrilines. The matrix shows all 91 possible pairs among these 14 mothers; 71 pairs contributed at least one dyad-year to the analysis (282 dyad-years total), with the remainder absent because the two mothers were never simultaneously followed in a year when both had a dependent offspring.

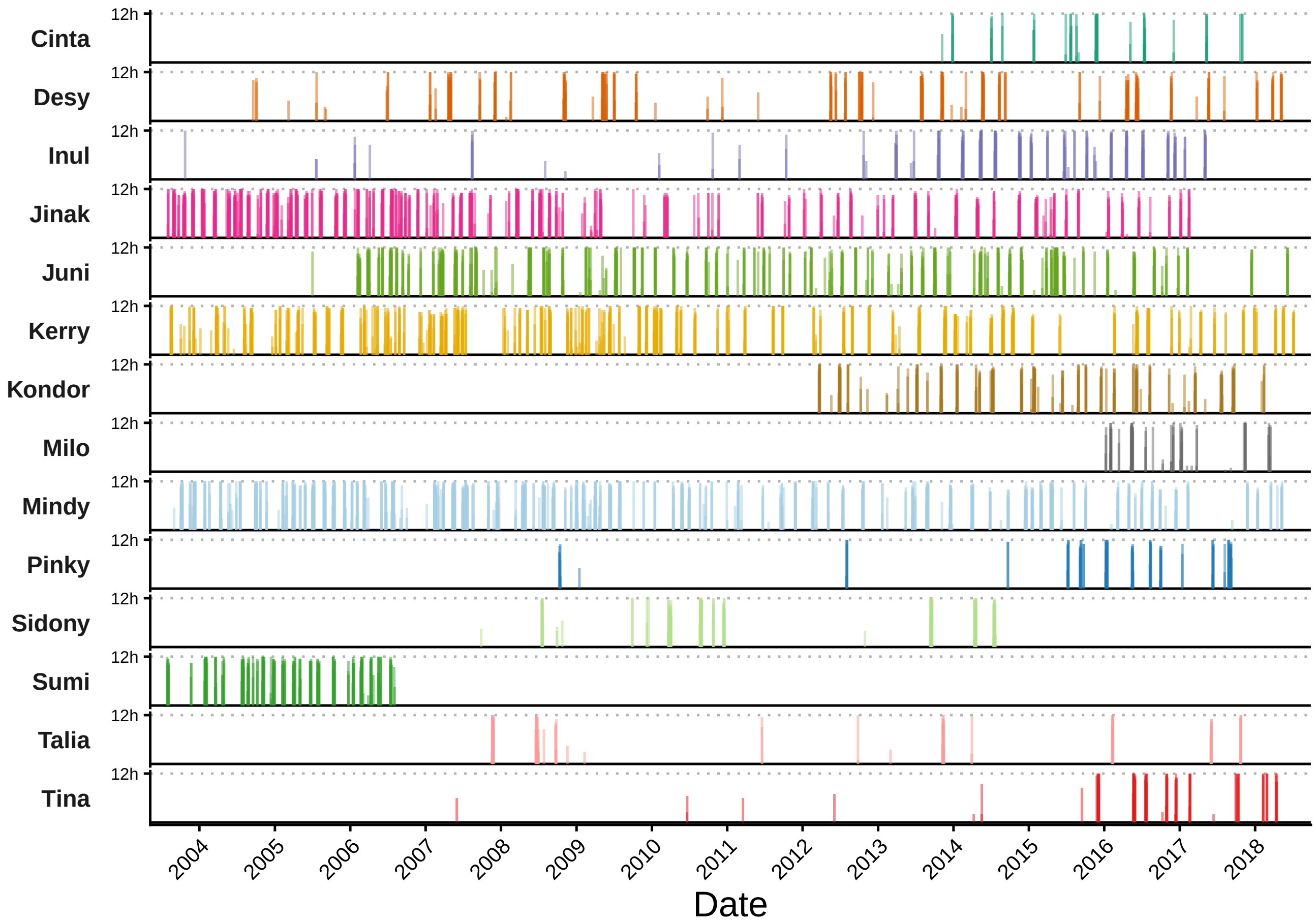

Fig. S2. GPS sampling coverage for focal orangutan mothers, 2003-2018. Each row shows daily tracking coverage for one female ($n = 14$) included in downstream analyses of daily path length and/or dyadic home-range overlap. Vertical bars indicate the proportion of a full follow day (12 h, equivalent to 24 fixes at 30-min sampling) covered on each date. Dotted horizontal lines mark full-day coverage. Coverage gaps reflect periods when females were not the focal subject of nest-to-nest follows.

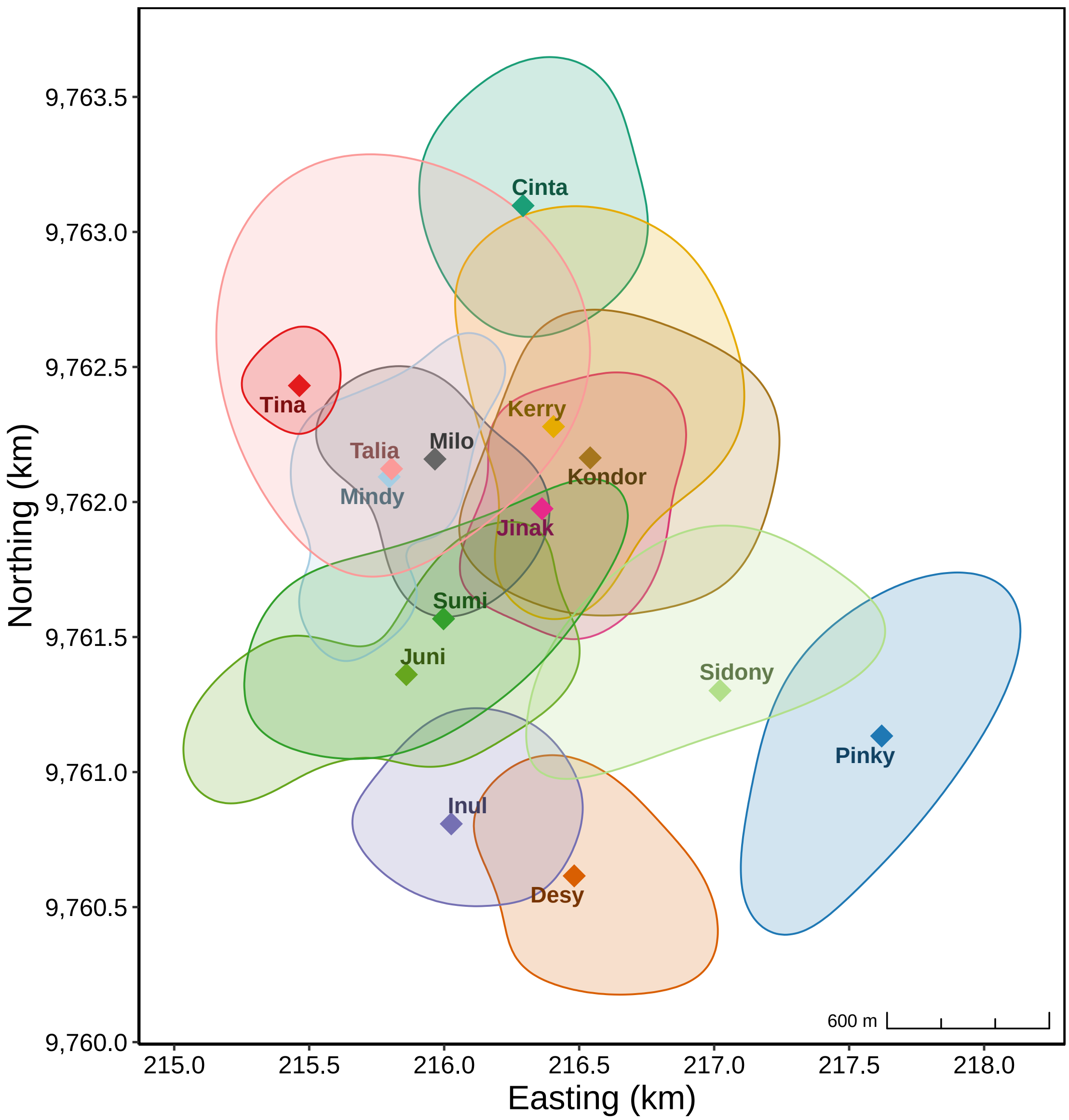

Fig. S3. Core areas and mean locations of focal orangutan mothers, 2003-2018. Polygons show each female's 50% core area, estimated from their full study-period autocorrelated kernel density estimate (AKDE). Diamonds mark each female's all-time mean location.

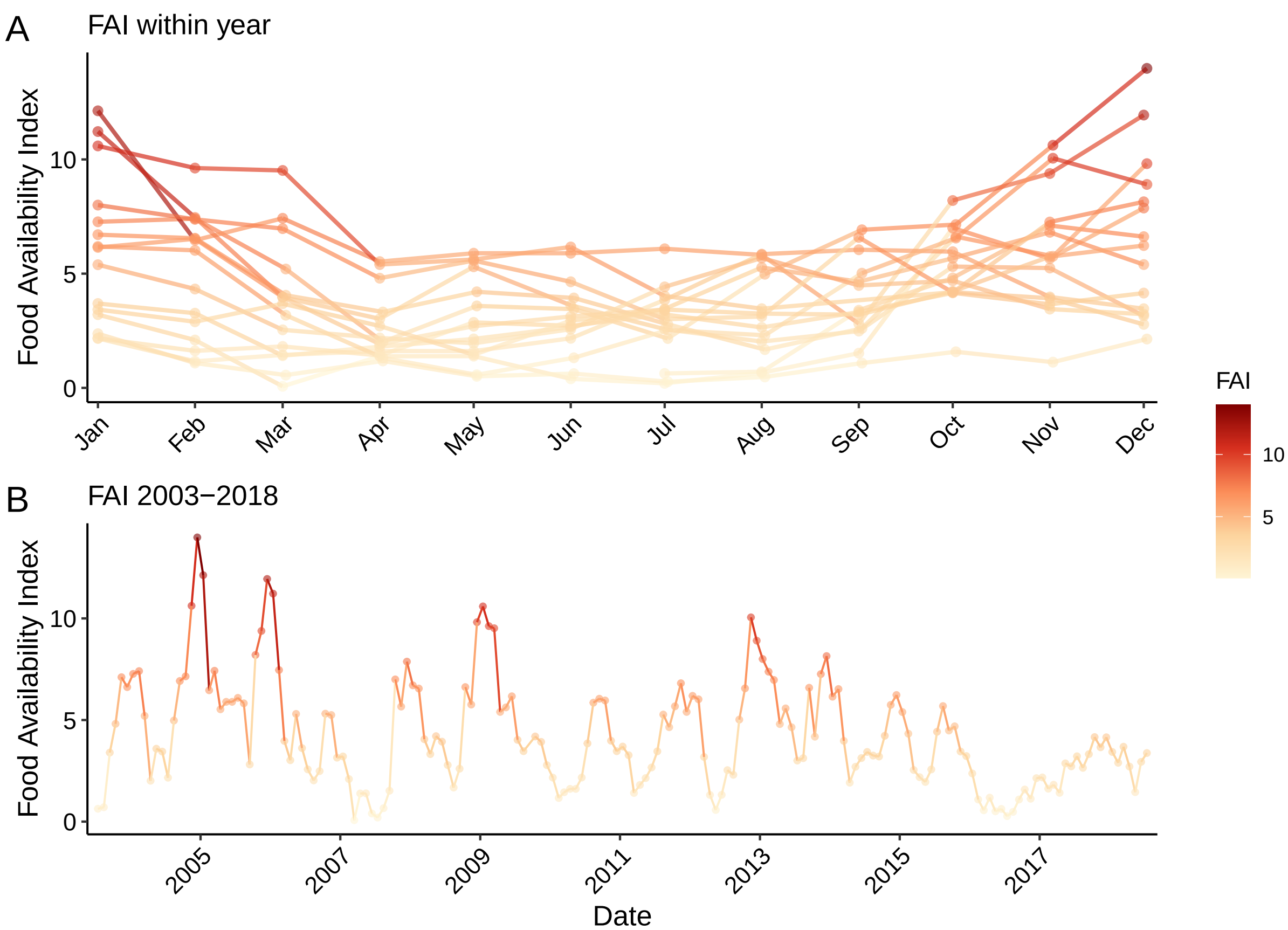

Fig. S4. Fruit Availability Index (FAI) at Tuanan, 2003-2018. FAI was scored monthly from phenological monitoring of marked trees along established transects at the field site. (*a*) Within-year seasonal variation: each line represents one calendar year. (*b*) Full monthly time series, 2003-2018. Colour reflects FAI value across both panels.

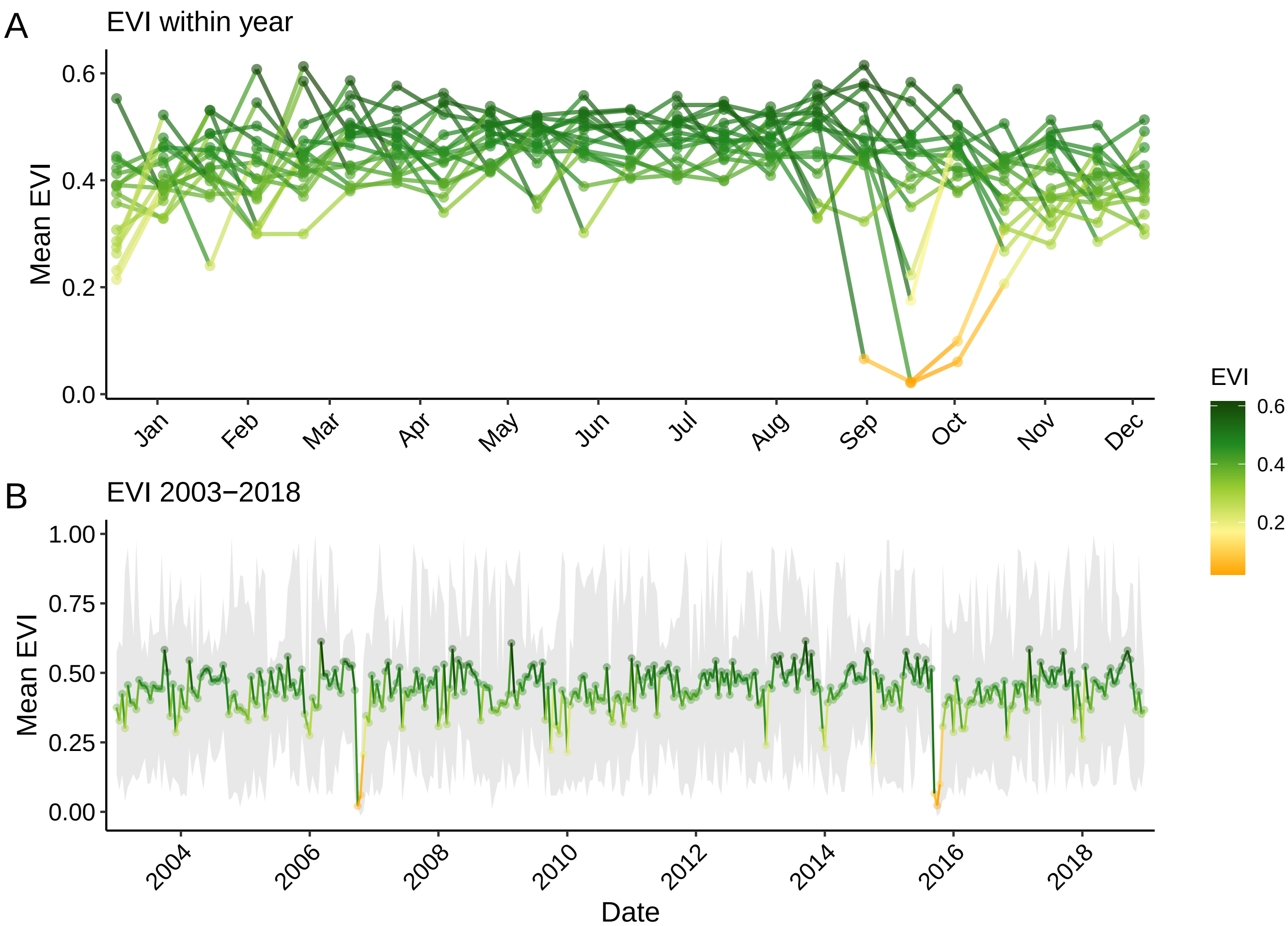

Fig. S5. Enhanced Vegetation Index (EVI) at Tuanan, 2003-2018. (*a*) Within-year seasonal variation: each line represents one calendar year, with points showing 16-day composite values. (*b*) Full time series, 2003-2018, showing the spatial mean across pixels (line and points) and the spatial range across the window (grey ribbon). Colour reflects EVI value across both panels.

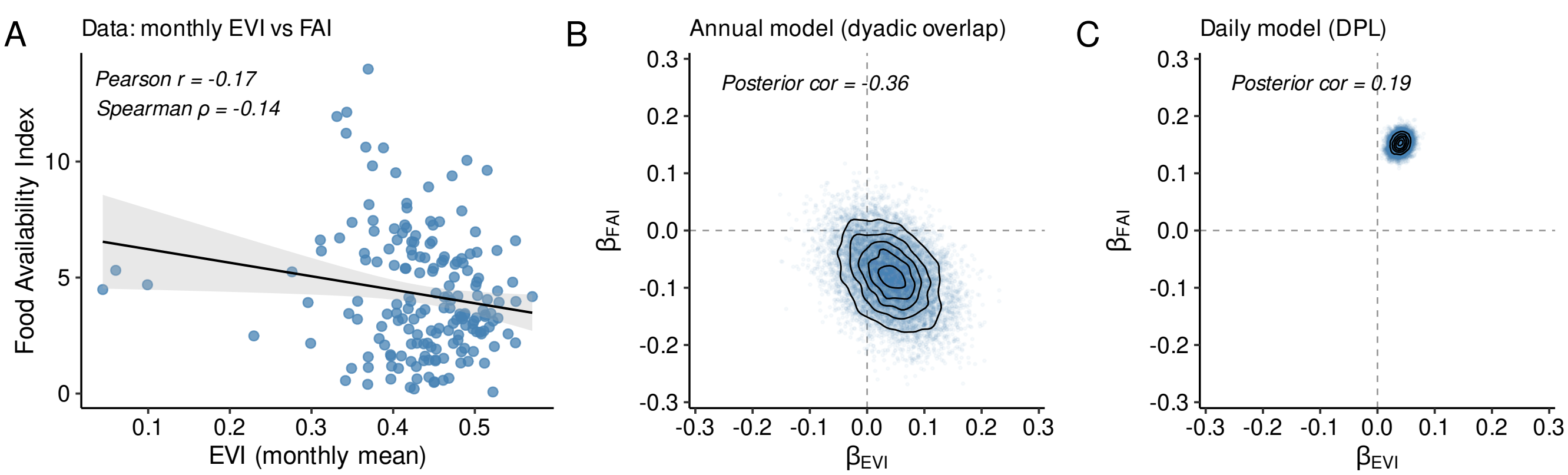

Fig. S6. EVI and FAI capture distinct sources of environmental variation. (*a*) Monthly mean EVI plotted against monthly habitat-wide FAI (2003-2018). The black line shows a linear regression with 95% confidence band; Pearson and Spearman correlations are shown for descriptive purposes only. (*b, c*) Joint posterior distributions of the standardized FAI and EVI regression coefficients in the annual dyadic-overlap model (*b*) and the daily path-length model (*c*). Blue points are individual posterior draws; black contours show 2D density. Dashed lines mark zero on each axis. The posterior correlation between the two coefficients is reported in each panel; roughly circular posteriors indicate that each effect was independently identifiable in the model.

Play adjacency classifications and directed-travel methods.

Play adjacency classifications. Each follow day was classified relative to play events as a play day (day-of), the day immediately before or after a play day (day-before, day-after), or a non-adjacent day (baseline). Play days took precedence over adjacent-day classifications, so the day-before and day-after categories capture only non-play days flanking play events. Days that fell within one day of play events on both sides (e.g., a non-play day with play occurring both yesterday and tomorrow) were excluded from the day-before and day-after categories to avoid ambiguity in their classification. These classifications are used in the daily path length, feeding, and directed-travel adjacency analyses.

Range core estimation. For each mother on each focal date, we defined her range core as the mean location of all GPS fixes collected during the preceding 365 days, excluding the focal date itself. We used this GPS-fix mean, rather than an AKDE-based core, because fitting a separate rolling AKDE for every mother-date combination was computationally impractical. To ensure stable estimates, we retained only (mother, date) pairs with $\geq$100 fixes spanning $\geq$3 unique weeks across $\geq$2 unique months within the 365-day window.

Travel vectors and within-day windows. For each candidate day, we computed the focal mother's net travel vector from her first GPS fix to her last fix of the day. For within-day windows on play days, we measured the focal mother's net travel vector from the first fix of the day to the first observed play scan (pre-play), and from the last play scan to the last fix (post-play). Within-day windows were retained only if they contained $\geq$3 fixes spanning $\geq$60 minutes with $\geq$30 m of net displacement.

Directional and distance measures. We then computed two quantities per record: (i) the angular difference between the net travel bearing and the bearing from the starting location to the reference core (partner's core for the Approach analysis; the focal mother's own core for the Return analysis), used as input to descriptive circular statistics; and (ii) the change in net distance to the reference core (distance from start to core minus distance from end to core, with positive values indicating net travel toward the core), used as the response variable in the Bayesian regressions described in Other dyadic and individual-level models.

Descriptive circular statistics. For each record we computed the angular difference between the focal mother's net travel bearing and the bearing from her starting location to the reference core. Day-before and within-day pre-play observations were pooled for the Approach, and within-day post-play and day-after observations were pooled for the Return. Per-phase summaries are the mean direction and mean resultant length ρ, ranging from 0 (uniform) to 1 (all observed directions identical), computed using the `circular` R package (15).

Sample size differences across analyses. The daily path length, feeding time, Approach, and Return analyses all draw from the same pool of nest-to-nest follow days but apply different retention criteria. Daily path length requires successful CTSD path-length estimation; feeding requires available behavioural scan data; missingness differs slightly between the two. The Approach and Return analyses additionally require a qualifying 365-day prior centroid for both mothers (see above), $\geq$50 m net daily displacement, $\geq$100 m starting distance to the reference core, and $\geq$200 m mode-separation between cores. These thresholds are required so that net travel direction can be meaningfully resolved. The Approach and Return baselines exceed those of the daily path length and feeding analyses because the dyad-indexed structure assigns one baseline row per focal-date per potential play partner (defined as any other mother whose infant was observed playing with the focal's infant at some point during the study; see Other dyadic and individual-level models). The rose-plot categories in Figure 3c-d also combine full-day adjacent observations with within-day pre- and post-play windows, exceeding the day-before/day-after counts in Figure 3a-b.

Causal inference framework and DAG specification. We framed each analysis as a question about causal effects rather than associations, and used directed acyclic graphs (DAGs) to make the assumed causal structure of the system explicit. A DAG encodes which variables we assume cause which others and lets us identify the minimal set of covariates needed to estimate a target causal effect without bias (16–18). We constructed our DAGs using the `dagitty` package (19) in `R`. We treat each DAG as an explicit, falsifiable set of assumptions about the system, drawn from biological reasoning rather than learned from the data.

Throughout this section we use a set of terms from the causal inference literature. The *exposure* is the variable whose effect we want to estimate (e.g., offspring age difference). The *outcome* is the variable that effect acts on (e.g., home-range overlap). An *estimand* is the specific causal quantity we aim to estimate, defined by an exposure, an outcome, and the variables held fixed. A *confounder* is a variable that causes both the exposure and the outcome and would bias the estimate if not adjusted for. A *mediator* is a variable through which part of the exposure's effect on the outcome is transmitted. A *backdoor path* is any non-causal path between the exposure and outcome that would

carry spurious association if left open. A *collider* is a variable caused by both the exposure and the outcome; unlike a confounder, adjusting for it *opens* a spurious path and introduces bias, so colliders are left unadjusted.

Our statistical models fall into two causal families. The dyadic models (home-range overlap and infant–infant play) share an exposure (offspring age difference within a mother–mother dyad) and a confounding structure (Figure S7). The individual-level models (daily path length and feeding time) share an exposure and a distinct confounding structure (Figure S8). We present one DAG per family.

Estimands. For the overlap models (BC overlap vs. intersection area), the estimand is the direct causal effect of offspring age difference on dyadic home-range overlap within a dyad-year. The two models share an estimand and a DAG and differ only in the outcome scale. For the infant–infant play model, the estimand is the *total* causal effect of offspring age difference on play. We estimate the total rather than direct effect because our key question is whether dyads with similar-aged offspring play more overall, integrating both the direct mechanism (infants of similar age preferring to play once encountered) and the indirect mechanism (mothers of similar-aged infants overlapping spatially and creating encounter opportunities). For the day-level models, the estimand is the direct causal effect of focal-day offspring play (i.e., "play day") on the focal's daily energetic outcome (daily path length and feeding time).

DAG for the dyadic models. The exposure is offspring age difference (OFF_AGE_DIFF); the outcomes are home-range overlap (OVER) and infant–infant play (PLAY). Observed covariates are mother–mother relatedness (REL), mean dyadic home-range area (HRA), combined offspring age (OFF_AGE_SUM), combined mother age (MAGE), fruit availability (FAI), and vegetation greenness (EVI). Latent nodes capture a landscape-level environmental state (ENV), an unobserved spatial food-distribution variable (DISTRIB), and residual unmeasured drivers of past reproductive timing ($U_{\text{OFF\_AGE\_DIFF}}$, e.g., maternal body condition and prior infant survival). The full set of arrows is shown in Figure S7.

We highlight five assumptions. First, REL → OFF_AGE_DIFF:kin dyads have non-random offspring-age structure as a demographic consequence of relatedness. Because kinship arises through birth timing, related females are themselves age-structured (e.g., mother-daughter pairs differ in age by at least the age at first reproduction; sisters by their mother's interbirth interval), which in turn constrains the age differences between their respective offspring. Unrelated dyads face no such constraint. This makes REL a confounder of the OFF_AGE_DIFF → OVER relationship and required in the adjustment set. Second, REL → OVER, PLAY: kin share space and tolerate proximity at higher rates (3). Third, OVER → PLAY: greater spatial overlap creates more opportunity for infant–infant encounter, making OVER a mediator on one of the paths from OFF_AGE_DIFF to PLAY. Fourth, MAGE causes OFF_AGE_DIFF, OFF_AGE_SUM, and HRA: a young mother cannot have an offspring age gap larger than her own reproductive history allows, mothers with longer reproductive histories tend to have older offspring, and older mothers occupy more established home ranges. This makes MAGE a confounder of the OFF_AGE_DIFF → OVER, PLAY relationships. Fifth, OFF_AGE_SUM and OFF_AGE_DIFF share unmeasured causes ($U_{\text{OFF\_AGE\_DIFF}}$, MAGE) but neither causes the other; both are functions of offspring birth dates rather than of each other. The remaining assumptions are as follows: ENV drives FAI, EVI, and DISTRIB; productivity and food distribution shape HRA; HRA, FAI, and EVI contribute to OVER; OFF_AGE_SUM contributes to PLAY directly and to HRA; and OFF_AGE_DIFF acts on OVER and PLAY.

Adjustment. For the direct effect of OFF_AGE_DIFF on OVER, there are two minimal sufficient adjustment sets: {EVI, FAI, HRA, REL} or {MAGE, OFF_AGE_SUM, REL}. These are equivalent for identification: backdoor paths can be closed either by intercepting environmental and ranging effects at HRA or by intercepting demographic effects upstream at MAGE and OFF_AGE_SUM. We adopt the first set for the overlap model and additionally condition on OFF_AGE_SUM for precision; none of these are descendants of OFF_AGE_DIFF, so this does not introduce post-treatment bias. For the total effect of OFF_AGE_DIFF on PLAY, the only minimal sufficient adjustment set is {MAGE, OFF_AGE_SUM, REL}. Unlike the overlap model, no alternative set based on HRA exists, because HRA does not lie on any backdoor path to PLAY in our DAG. The play model conditions on OFF_AGE_SUM (entered with a quadratic term to capture nonlinearity in developmental stage) and REL, and additionally on FAI and EVI for precision. We do not include MAGE in the primary models for two reasons (see *Maternal age* below).

Maternal age. The DAG identifies MAGE as a confounder of OFF_AGE_DIFF → (OVER), (PLAY). We do not include it as a fixed effect in the primary models for two reasons. First, stable between-mother variation in mother age is fully absorbed by the multi-membership random intercept on mother identity. The remaining unhandled component is within-mother aging across the study period, which is uniform across mothers (all aged ∼15 years together over 2003–2018) and is largely captured by OFF_AGE_SUM, which tracks the same temporal axis. Second, mothers' birth years are uncertain: eleven of fourteen mothers were already adults when observation began in 2003. Their birth years were reconstructed by working backward from the youngest known offspring using estimated interbirth

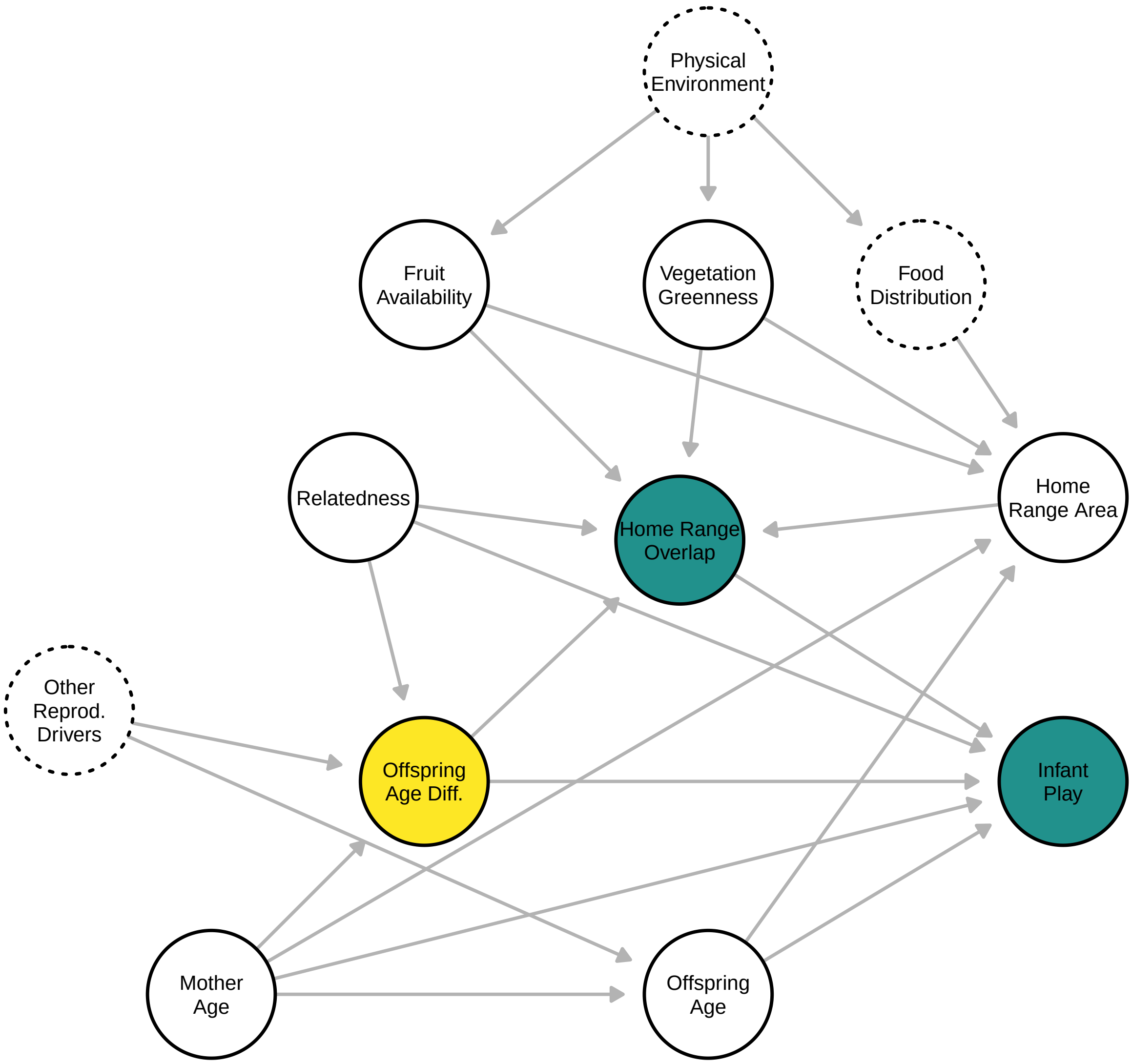

Fig. S7. Directed acyclic graph for the dyadic models (home-range overlap, intersection area, and infant-infant play). Solid circles denote observed variables; dashed circles denote latent variables. Yellow: exposure (OFF_AGE_DIFF). Teal: outcomes (OVER, PLAY); the same DAG applies to intersection area. See SI Materials and Methods for variable definitions, arrow justifications, and adjustment sets.

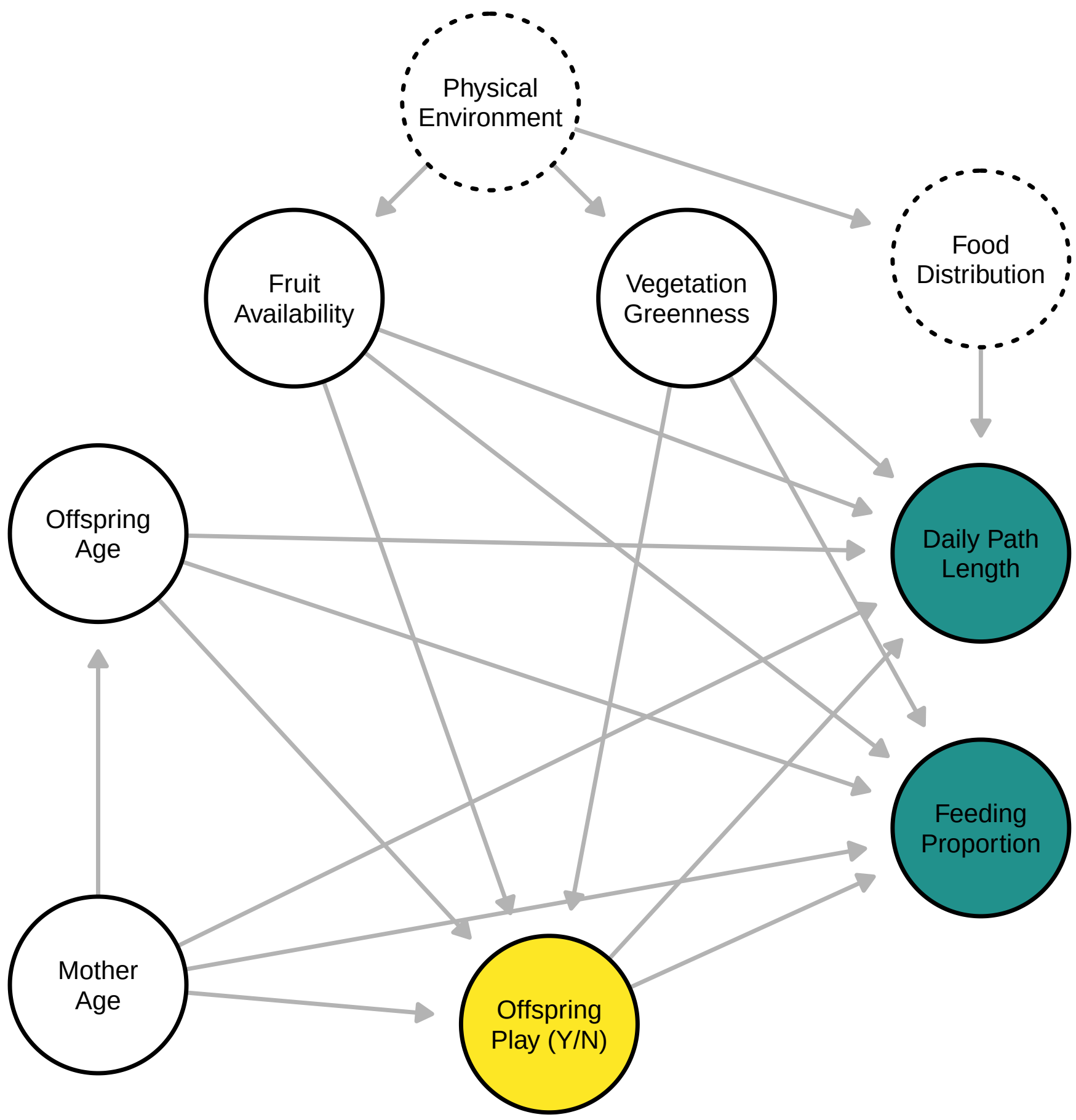

Fig. S8. Directed acyclic graph for the day-level models (daily path length and feeding proportion). Solid circles denote observed variables; dashed circles denote latent variables. Yellow: exposure (PLAY). Teal: outcomes (DPL, FEED). See SI Materials and Methods for variable definitions, arrow justifications, and adjustment sets.

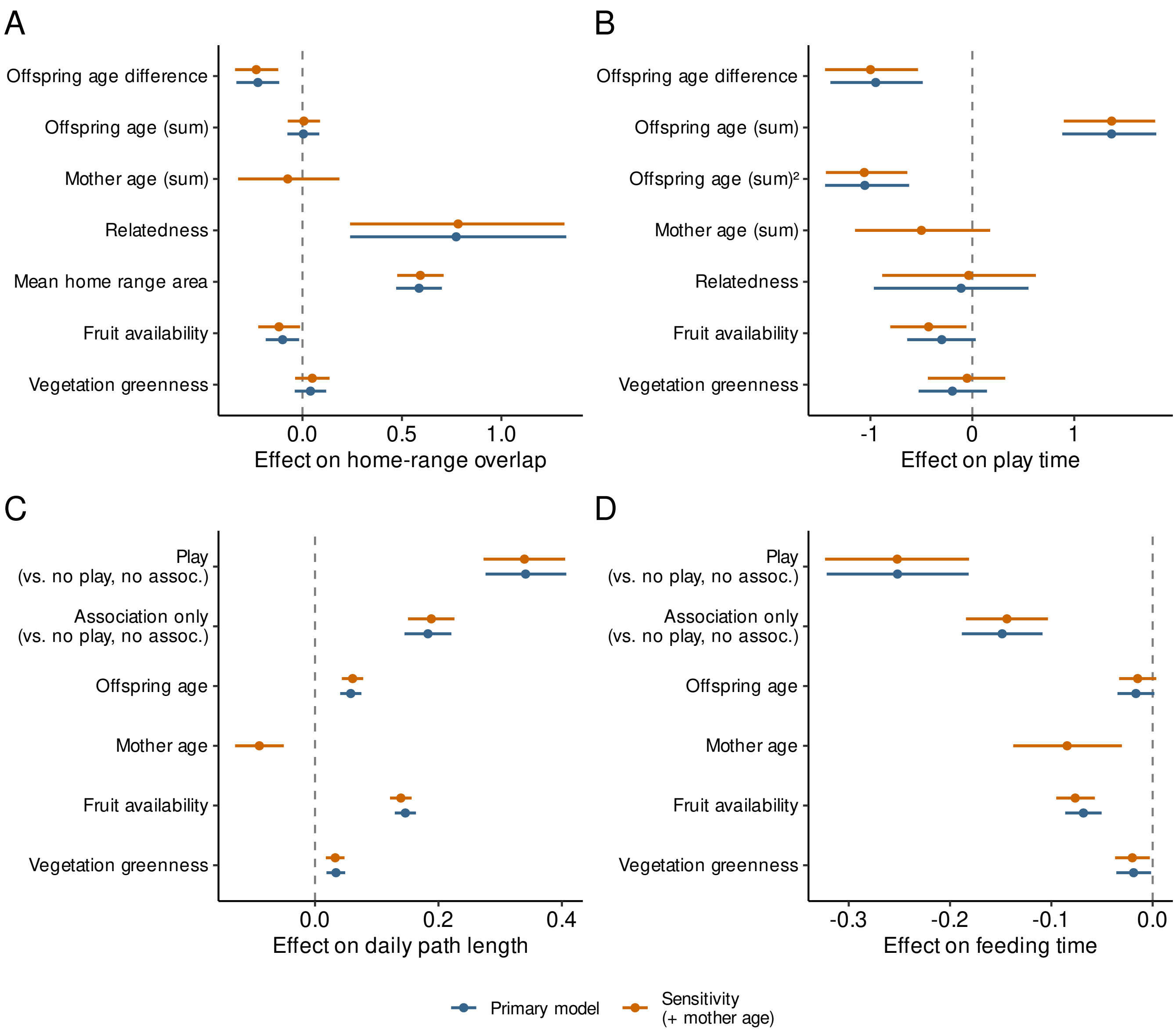

Fig. S9. Sensitivity of dyadic- and individual-level effects to the inclusion of maternal age. 89% posterior point intervals for fixed-effect coefficients in the primary models (blue) and sensitivity models (orange) for (a) home-range overlap, (b) infant–infant play time, (c) daily path length, and (d) feeding time. Sensitivity models include summed maternal age (a, b) or maternal age (c, d) as an additional covariate, entered via a measurement-error specification (20) that propagates encoding uncertainty in birth-year estimates (most mothers had only upper-bound birth years; see Methods). For the play model (b), coefficients shown are from the count component of the hurdle, describing effects on the rate of play conditional on any play occurring.

intervals and age at first reproduction, yielding minimum-age estimates that underestimate true age by an unknown individual-specific amount (older sons that dispersed before observation began, infants that did not survive, daughters that emigrated to areas outside the study). To verify robustness, we report sensitivity analyses (Figure S9) in which mother age is included via a measurement-error specification (20) that propagates encoding uncertainty into the posterior. The focal offspring age difference effect remained directionally consistent across all specifications.

DAG for the day-level models. The exposure is offspring social play on the focal day (PLAY). The outcomes are daily path length (DPL) and the proportion of scans spent feeding (FEED). Observed covariates are the focal mother's offspring age (OFF_AGE), mother age (MAGE), FAI, and EVI; ENV is again the latent parent of the observed environmental proxies (Figure S8).

The arrows we assume are: OFF_AGE → PLAY (offspring engage in social play differently across developmental stages); OFF_AGE → DPL, FEED (maternal travel and feeding patterns vary with offspring developmental stage, as mothers adjust the pace of travel to their offspring's locomotor capacity and adjust feeding effort to changing energetic demands of dependence); MAGE → OFF_AGE (younger mothers have younger offspring), and MAGE → PLAY, DPL, FEED (mother age may independently affect activity budgets, ranging, and social behavior); FAI, EVI → PLAY, DPL, FEED (productivity and phenology shape both the opportunity for play and the travel and feeding required to meet daily energetic needs); FAI, EVI, DISTRIB → DPL (food distribution shapes how far mothers must travel to access resources); and PLAY → DPL, FEED (mothers monitoring offspring as they play takes time away from feeding which could have downstream effects on daily travel; mothers may also have to travel further to find suitable play partners).

Adjustment. The minimal sufficient adjustment set for the direct effect of PLAY on either outcome is {EVI, FAI, MAGE, OFF_AGE}. We condition on OFF_AGE, FAI, and EVI directly. We verify in sensitivity analyses (Figure S9) that explicitly including MAGE as a measurement-error covariate does not qualitatively alter the results.

Assumptions and limitations. For the dyadic models, our DAG cannot rule out the possibility of unmeasured time-varying confounders that affect both reproductive timing and ranging behavior. We also assume FAI and EVI together adequately proxy the latent environmental state; because they capture distinct aspects of productivity (ground-based fruit vs. remote-sensed canopy greenness) and are not collinear in our data; we consider this defensible but imperfect.

For the day-level models, plausible unmeasured day-level confounders include weather, chance encounters with neighbouring mothers, and within-day maternal state. We further assume the PLAY → DPL arrow runs in the direction we have drawn it, that mothers' elevated travel reflects pursuit of play opportunities rather than play being an incidental by-product of travel undertaken for other reasons.

DAG considerations for the directed-travel models. The Approach and Return models analyze a day-level exposure (play-adjacent phase) and an individual-level response (the focal mother's change in distance to the reference core — the partner's core for Approach, the focal's own core for Return). Because each row relates to a specific (focal, partner) dyad, both models use the same adjustment set as the dyadic models (relatedness, summed offspring age, offspring age difference, FAI, and EVI; Figure S7). Mode separation (distance between focal and partner cores), daily path length, and observation-window duration are included to capture geometric and sampling features that likely affect approach and return distances but are not part of the DAG.

Statistical formulation of the dyadic home-range overlap model. We modelled the annual pairwise home-range overlap between mothers as a symmetric social relations model (SRM) (21–23), in which the dyadic outcome is decomposed into individual-level contributions and a dyad-specific component. Because our home-range overlap metric is symmetric by construction (the overlap between mothers i and j is identical to the overlap between j and i), we used a symmetric SRM in which both members of a dyad contribute through a single shared random effect on individual identity, implemented as a multi-membership term (20). This contrasts with asymmetric SRMs (e.g., for directed interactions) in which each individual contributes separately as "actor" and "partner" through correlated varying effects.

The Bhattacharyya coefficient (BC) of overlap is bounded on $[0, 1]$, and each estimate $\widehat{\mathrm{BC}}_{ij,t}$ for dyad $\{i, j\}$ in year t has an associated 95% confidence interval derived from the autocorrelated kernel density estimation procedure (6). To incorporate this estimation uncertainty into the model and to retain a tractable Gaussian likelihood, we logit-transformed each point estimate and its bounds, computed the standard error of the logit estimate from the transformed CI width as $\sigma_{ij,t} = (\mathrm{logit}(\widehat{\mathrm{BC}}^{U}_{ij,t}) - \mathrm{logit}(\widehat{\mathrm{BC}}^{L}_{ij,t}))/3.92$, and modelled the logit-transformed estimate with a measurement-error term (20).

The full specification is:

$$y_{ij,t} \sim \text{Normal}(\mu_{ij,t},\ \sigma_{ij,t}) \quad [1]$$

$$\mu_{ij,t} = \bar{\alpha} + d_{\{ij\}} + \tfrac{1}{2}(m_i + m_j) + \beta_D\, D_{ij,t} + \beta_O\,(O_{i,t} + O_{j,t}) + \beta_R\, R_{ij} + \beta_H\, H_{ij,t} + \beta_F\, F_t + \beta_E\, E_t \quad [2]$$

where $y_{ij,t} = \text{logit}(\widehat{\text{BC}}_{ij,t})$ is the logit-transformed overlap estimate for dyad $\{i, j\}$ in year t, and $\sigma_{ij,t}$ is its known measurement standard error. The linear predictor $\mu_{ij,t}$ comprises:

- $\bar{\alpha}$: the overall intercept, representing the mean logit overlap across all dyads and years;
- $d_{\{ij\}}$: a dyad-level varying intercept capturing the unique pairwise deviation of dyad $\{i, j\}$ from the individual-level expectations;
- m_i, m_j: individual-level varying intercepts for the two mothers in dyad $\{i, j\}$, entered through the multi-membership structure $\frac{1}{2}(m_i + m_j)$, which assigns equal weight to both mothers and reflects the symmetric contribution of each individual to dyadic overlap;
- $D_{ij,t}$: absolute difference in offspring ages within the dyad in year t;
- $O_{i,t}, O_{j,t}$: the ages of mother i's and mother j's focal offspring in year t, entered as a single summed term $(O_{i,t} + O_{j,t})$ with a shared slope β_O, reflecting the symmetric contribution of each offspring's developmental stage to dyadic outcomes;
- R_{ij}: pedigree-based coefficient of relatedness between mothers i and j;
- $H_{ij,t}$: mean home-range area of the two mothers in year t;
- F_t, E_t: mean fruit availability index (FAI) and Enhanced Vegetation Index (EVI) over the dyad-year sampling window.

The varying intercepts are modeled as independent draws from zero-centered normal distributions:

$$d_{\{ij\}} \sim \text{Normal}(0,\ \tau_d) \quad [3]$$

$$m_i \sim \text{Normal}(0,\ \tau_m) \quad [4]$$

where τ_d and τ_m are the among-dyad and among-mother standard deviations, respectively.

We placed weakly informative priors on all parameters (17):

$$\bar{\alpha} \sim \text{Normal}(-0.55,\ 0.5) \quad [5]$$

$$\beta_D, \beta_O, \beta_R, \beta_H, \beta_F, \beta_E \sim \text{Normal}(0,\ 1) \quad [6]$$

$$\tau_d, \tau_m \sim \text{Exponential}(1) \quad [7]$$

The intercept prior was centred on the empirical mean of the logit-transformed BC estimates (≈ -0.55); and slope priors are weakly regularising on the standardised scale.

Symmetric SRM structure and its multiple membership implementation. Because our response is symmetric, we did not estimate the actor-partner correlation that is standard in asymmetric SRMs. Each individual contributes a single varying intercept through both "slots" of every dyad it participates in, and partial pooling across dyads that share an individual is achieved directly through the multi-membership structure rather than through a correlated bivariate random effect.

The specification above may be described as a multiple membership relations model (24), and is functionally equivalent to a symmetric (non-directed) SRM coded directly in a probabilistic programming language such as `Stan`; the two differ only in how the individual effects are constructed. A symmetric social relations model lacks a scalar or weight parameter that is multiplied by the individual-level varying effect as has been mathematically formalized (mostly in the supplementary material) in (23–26). Coded directly, individuals are referenced by identity and the two members of a dyad enter as a sum of their effects, each with an implied coefficient of 1.

The multiple-membership model is functionally the same, but includes a weight value that the user can specify (27, 28), and is commonly used in various social network approaches (albeit with different weights or scalars)— see

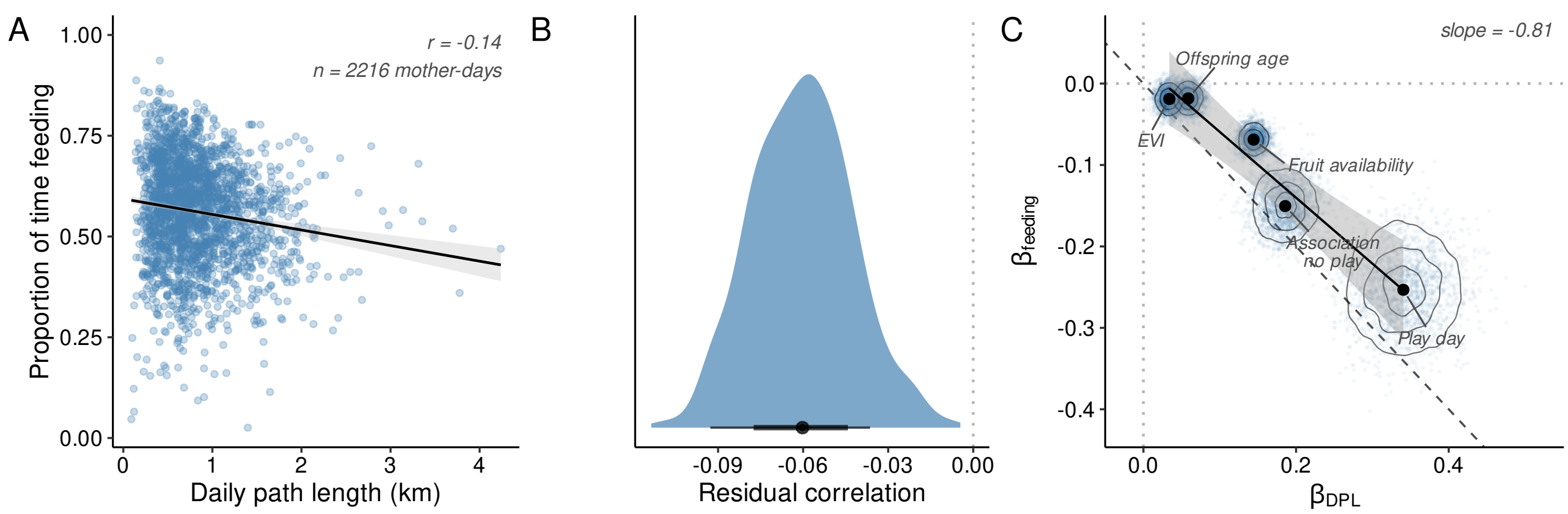

Fig. S10. Daily path length and feeding rate are coupled but capture partly independent aspects of mothers' activity budgets. *(a)* Daily proportion of time spent feeding plotted against daily path length, with one point per mother-day ($n = 2216$). The black line and grey ribbon show a linear regression with a 95% confidence band. The two outcomes are weakly negatively correlated ($r = -0.14$). *(b)* Posterior distribution of the residual correlation between the two model fits, computed by correlating posterior residuals across mother-days within each posterior draw (median $r = -0.06$, 89% HDI $[-0.09, -0.04]$). *(c)* For each predictor that appears in both models, the joint posterior of its DPL coefficient (β_{DPL}, x-axis) against its feeding coefficient ($\beta_{feeding}$, y-axis), shown as 2D density contours with black points at the posterior medians. The dashed grey line marks $y = -x$, where the points would fall if the two models carried identical information with opposite signs. The solid blackline and grey ribbon show a linear regression with a 95% confidence band through the five coefficient medians (slope $= -0.81$). All five coefficients sit above the $y = -x$ reference, meaning that for every predictor, daily path length shifts more than feeding rate does.

(29, 30). `brms` instead uses a classic multiple membership model (27) which defines varying effects on data-frame columns, so each individual is split across the two member columns of a dyad; the multiple-membership term recombines these columns into a single per-individual effect and normalizes the membership weights to sum to one, so that each member enters with weight $\frac{1}{2}$ and the dyad's contribution is an average rather than a sum. This rescales the among-mother standard deviation τ_m by a constant factor but leaves predictions unchanged. In both formulations, the relationship effect is the dyad-level varying intercept $d_{\{ij\}}$. These model types are isomorphic and functionally make the same predictions– they just share different preferences for the use and/or magnitude of normalization constants.

Other dyadic and individual-level models. The other models in this study share components of the structure above and we describe them by their differences. The infant-infant play model retains the dyadic varying-effects structure (multi-membership term on mother identity and dyad-level varying intercept) and the same set of covariates (offspring age difference, summed offspring age, relatedness, FAI, and EVI), but is fitted to annual play-scan counts $N_{ij,t}$ using a hurdle negative binomial likelihood (log link). The log of joint focal-scan observation effort is included as an offset, and a quadratic term in summed offspring age ($\beta_{O^2}\,(O_{i,t} + O_{j,t})^2$) is added to both the count and the hurdle components, reflecting the non-monotonic developmental trajectory of play previously documented in this population (31). The hurdle component shares the same linear predictor as the count component but on the logit scale, with observation effort entered as a covariate rather than as an offset.

The models for daily path length and feeding time use individual-level structure: a single varying intercept per focal mother and focal offspring age, FAI, and EVI as covariates. The exposure variable was operationalized in two complementary specifications: a three-level predictor (alone with offspring; association without play; infant-infant play day) and a four-level adjacency predictor (no-play; day-before play; day-of play; day-after play). Daily path length is modelled with a Gamma likelihood (log link) and a measurement-error term derived from CTSD confidence intervals, and feeding effort with a beta-binomial likelihood (logit link) on counts of feeding scans out of total scans per follow day.

The Approach and Return models share a structure that differs from the dyadic and day-level models above. Rows are indexed at the (focal mother, partner, date) level, with a Gaussian likelihood on the change in distance to the reference core (partner's core for Approach, focal's own core for Return). With this dyad-indexed structure, each baseline focal-date contributes one row per potential play partner (the set of dyads in which the focal mother has been observed playing). This builds a counterfactual baseline distribution of the focal's daily travel for each (focal, partner) pair — what her travel looks like, relative to that specific partner's core, on days when no play is impending. The dyad random intercept accounts for non-independence among the multiple baseline observations sharing a focal-date by partially pooling within each dyad. The predictor of interest is a categorical phase variable contrasting baseline days with day-before and within-day pre-play windows (Approach) or with within-day post-play and day-after windows (Return). Covariates follow the DAG-based adjustment set described above (see DAG considerations for the directed-travel models), with random intercepts for focal mother and mother-dyad to account for repeated sampling within individuals and dyads. Although the dyad structure is reflected through the random intercept, the response measures only the focal mother's travel, with the partner serving as a geometric reference point rather than as a second actor (note: simultaneous nest-to-nest tracking of both mothers in a dyad is rarely available in the data).

Supplementary Results

Offspring independently contribute to the age-difference effect on play. The play model presented in the main text uses total observation effort as the offset, estimating the total effect of offspring age difference on play frequency per unit of observation time (i.e., play rate). This estimand encompasses both mechanisms by which age difference might affect play: the indirect path through which mothers of similar-aged infants are in association more (creating opportunity for play), and the direct path through which similar-aged infants engage more once together in association. Note that association here plays the same role that home-range overlap plays in the dyadic DAG (Figure S7): both index the extent to which mothers create opportunity for their offspring to encounter one another. To isolate the second mechanism, we refit the same model structure with joint-association scans (where both mothers and their dependent infants were observed together) as the offset. The age-difference coefficient under this offset estimates the play rate per scan of opportunity for the infants to play, rather than per scan of observation time. The effect remained strongly negative under both specifications ($\beta_{\text{association}}$ = -0.89 [-1.36,-0.41]; $PP > 0 = 0.003$; $\beta_{\text{observation}}$ = -0.95 [-1.41,-0.51]; $PP > 0 = 0.002$; Figure S11a). The persistence and stability of the age-difference effect across these specifications indicates that offspring themselves contribute substantively to the pattern: similar-aged infants engage in more play once their mothers have brought them into proximity.

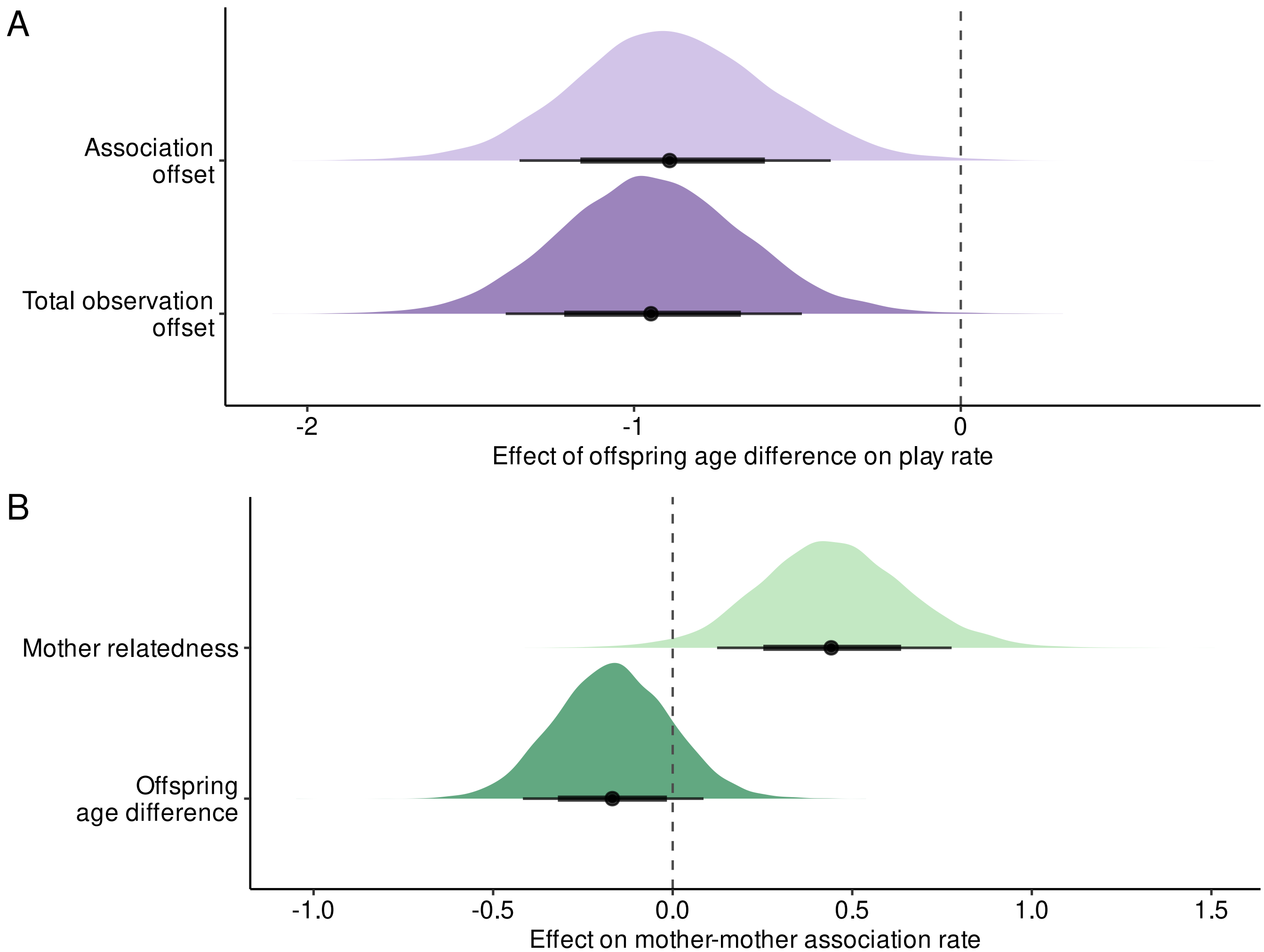

Fig. S11. Decomposition of the age-difference effect on play rate. *(a)* Posterior estimates of the offspring age-difference coefficient in the play-scan model under two offset specifications. Using total observation scans as the offset estimates the total effect of age difference on play, whereas using association scans conditions on the joint presence of the two mothers and their dependent offspring. *(b)* Posterior estimates of the mother relatedness and offspring age-difference coefficients in the mother-mother association model. Points show posterior medians, and thick and thin lines show 66% and 89% credible intervals, respectively.

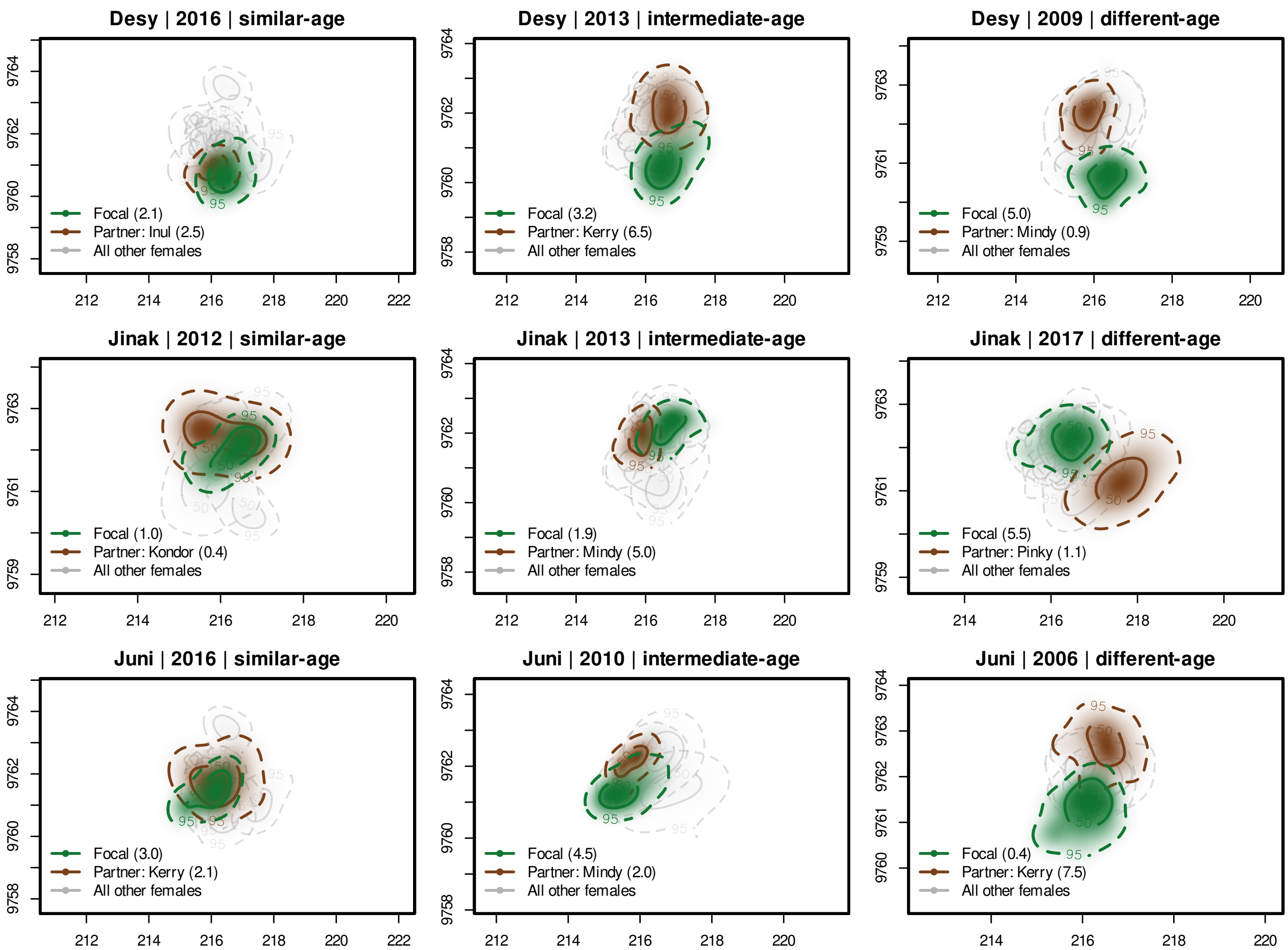

Fig. S12. Examples of annual home-range overlap varying with offspring age similarity. Selected dyad-years illustrating the spatial pattern underlying the results in Figure 1. Each row shows a focal mother (green) paired with three different neighbors (brown) in separate dyad-years representing similar (left column), intermediate (middle column), and large (right column) offspring age differences. Shaded regions show 95% and 50% autocorrelated kernel density estimate (AKDE) contours, with underlying color gradients indicating the probability of space use. Light grey contours show home ranges of all other mothers in the study population for that year. Offspring ages for each dyad are shown in the panel legends.

Mothers independently contribute to the age-difference effect on play. The previous analysis isolates the offspring contribution but does not isolate the maternal contribution to the age-difference effect on play. Two further lines of evidence point to a substantial maternal contribution operating through space use. First, the main-text home-range overlap result shows that mother-mother spatial overlap (Bhattacharyya coefficient) is credibly higher for dyads with similar-aged offspring, indicating that mothers structure their intensive space use to bring similar-aged offspring into closer spatial proximity. Second, we fitted a hurdle negative-binomial symmetric SRM with mother-mother association scans as the outcome, joint observation effort as the offset, and the same environmental and demographic covariates as the other dyadic models. Mother relatedness had a credibly positive effect on association rate (β = 0.43 [0.12, 0.76]; $PP > 0 = 0.984$; Figure S11b), replicating the kin-biased association pattern reported by (3). Offspring age difference showed a weakly negative effect (β = -0.18 [-0.42, 0.08], $PP > 0 = 0.126$) independent of mother relatedness. Third, we show in the main text that mothers oriented travel toward the partner's core on the day before play. Together, these findings suggest that mothers play an active role in shaping the social environment of their offspring by adjusting their broader ranging patterns. Moreover, it is unlikely that infants drive the ranging patterns given the nature of infant dependence in this population: infants are mostly carried during sustained travel up to about four years of age and are still carried intermittently (especially when crossing large gaps between trees) until around six years. All dependents remain within ∼50 m of their mothers (32), and a mother can veto an infant's approach to another female by moving in the opposite direction, forcing the dependent to follow.

Disentangling the costs of play from maternal association. To determine whether energetic costs reflected play itself or association with another mother, we refit the daily path length and feeding-time models with a four-level predictor distinguishing days with no association, days with non-mother association only, days with mother-mother association but no play, and play days (Figure S13). Daily travel on mother-mother association days without play was comparable to days with non-mother associates (β = 0.03 [-0.05, 0.10]; $PP > 0 = 0.708$), but mothers travelled credibly farther on play days than on either mother-mother association days (β = 0.14 [0.04, 0.22]; $PP > 0 = 0.992$) or other-association days (β = 0.16 [0.09, 0.24]; $PP > 0 = 1.000$), consistent with mothers actively travelling farther to arrange infant play encounters. Feeding costs were more closely tied to maternal association. Mothers fed less on mother-mother association days than on other-association days (β = -0.08 [-0.16, -0.01]; $PP > 0 = 0.046$), and feeding declined further on play days, although the contrast between play and mother-mother association days remained uncertain (β = -0.04 [-0.15, 0.05]; $PP > 0 = 0.248$). Together, these results suggest that elevated daily travel is specific to play, whereas reduced feeding is more closely tied to association with another mother, the social context that makes infant play possible. The weak additional reduction on play days may reflect time or attention diverted toward monitoring active play bouts, although this interpretation remains tentative.

Sensitivity to mixed association status in play-adjacency models. The play-adjacency models in the main text use a "no play" reference category that includes both fully-alone days and days when the focal mother associated with a non-offspring conspecific without infant play. Because non-offspring association can itself elevate travel and reduce feeding, we conducted a sensitivity analysis in which the no-play reference and the day-before/day-after categories were restricted to no-association days. Day-of-play observations are association days by definition and were unaffected. Within the original sample, the proportion of association days was 25.9% for the no-play reference, 27.7% for day-before, and 41.2% for day-after; the day-before category was therefore comparable to the reference, while day-after contained somewhat more association days. The filtered model (Tables S1 and S2) yielded effects in the same direction and of comparable magnitude to the original, including for day-after, supporting the interpretation that the adjacency gradient is not an artifact of association mixed into the "non-play day" categories. We retain the original specification in the main text because it preserves the full sample of adjacency days and is consistent with the temporal scale of the question (whether the days surrounding play differ from ordinary days), while the filtered model serves as a robustness check.

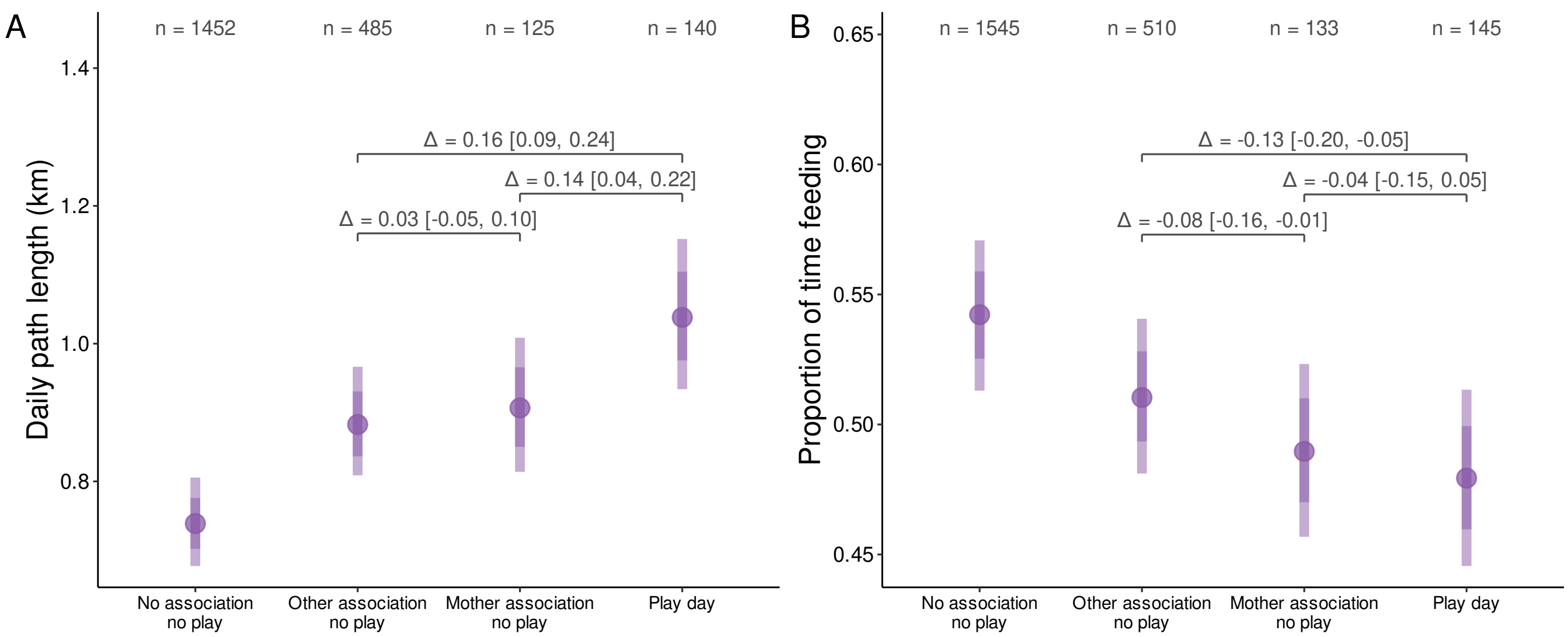

Fig. S13. Disentangling the costs of play from maternal association. Posterior predictions from Bayesian GLMMs of daily path length (*a*) and proportion of time feeding (*b*), comparing four categories: days when the focal mother was alone with her offspring (no association, no play; reference), days when she associated only with non-mother conspecifics without infant play (other association, no play), days when she associated with another mother without infant play (mother association, no play), and days when infant-infant play occurred (play day; which by definition involves mother-mother association). Posterior predicted values across categories are shown with covariates held at their mean values; sample sizes (*n*) are shown for each category. Points and dark/light lines show posterior medians with 66% and 89% credible intervals. Brackets and labelled values within each panel indicate the posterior median and 89% credible interval of the contrast between play days and the two association-only categories.

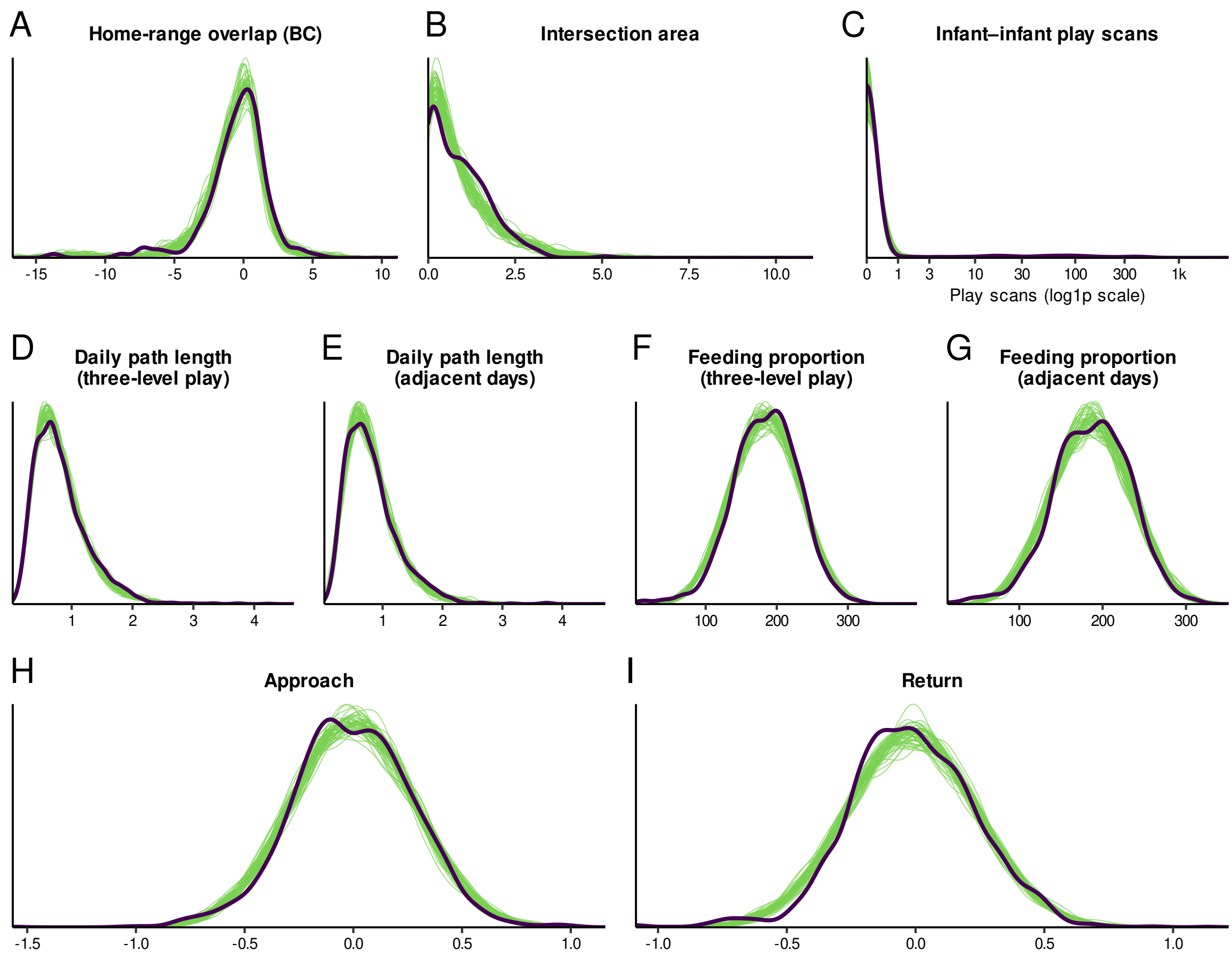

Fig. S14. Posterior predictive checks for all primary Bayesian models. Panels *a*, *b*, and *d*-*i* show the observed outcome distribution (dark purple line) overlaid with 50 draws from the posterior predictive distribution (green lines). Panel *c* shows a posterior-predictive bar plot with log-transformed y-axis rather than a density overlay because the play-scan outcome is a discrete count with substantial zero-inflation; observed counts at each value are shown as dark purple bars with posterior-predictive medians and intervals overlaid in green.

Sensitivity summaries for the play-adjacency models. The following tables compare the original and filtered specifications of the play-adjacency models (see SI Results for details).

Table S1. Sensitivity comparison of the daily path length adjacency model. Original (orig.) includes association days; filtered (filt.) restricts day_before and day_after to no-association days. Columns: posterior median with 89% HDI, $P(\beta > 0)$, sample size, and % association days in the original sample.

Category	β (orig.)	$P(\beta > 0)$ orig.	n (orig.)	% assoc.	β (filt.)	$P(\beta > 0)$ filt.	n (filt.)
Day before play	0.09 [-0.02, 0.20]	0.90	46	28.3	0.09 [-0.04, 0.22]	0.86	33
Day of play	0.34 [0.27, 0.41]	1.00	139	100.0	0.40 [0.33, 0.47]	1.00	139
Day after play	0.14 [0.04, 0.23]	0.99	70	42.9	0.23 [0.12, 0.36]	1.00	40

Table S2. Sensitivity comparison of the feeding-proportion adjacency model. Columns as in Table S1.

Category	β (orig.)	$P(\beta > 0)$ orig.	n (orig.)	% assoc.	β (filt.)	$P(\beta > 0)$ filt.	n (filt.)
Day before play	0.03 [-0.09, 0.15]	0.67	49	26.5	0.04 [-0.09, 0.18]	0.68	36
Day of play	-0.23 [-0.30, -0.16]	0.00	145	100.0	-0.28 [-0.35, -0.20]	0.00	145
Day after play	-0.04 [-0.13, 0.06]	0.28	75	42.7	-0.00 [-0.13, 0.12]	0.49	43

Posterior summaries for all models. The following tables provide posterior medians, 89% highest density intervals (HDIs), $\hat{R}$, and bulk and tail effective sample sizes (ESS) for all parameters in each of the nine primary Bayesian models reported in this study.

Table S3. Posterior summaries for the home-range overlap (BC) model. Median, 89% highest density interval (HDI), $\hat{R}$, and bulk and tail effective sample sizes (ESS) for each parameter.

Parameter	Median	HDI lo	HDI hi	Rhat	ESS bulk	ESS tail
Intercept (count)	-0.71	-1.28	-0.11	1	12316	8602
β Offspring age difference	-0.22	-0.33	-0.12	1	16561	9834
β Mean home-range area	0.59	0.47	0.70	1	16419	8443
β Offspring age (sum)	0.00	-0.07	0.09	1	18251	9929
β Relatedness	0.77	0.24	1.32	1	5321	6833
β Fruit availability	-0.10	-0.18	-0.01	1	15886	10009
β Vegetation greenness (EVI)	0.04	-0.04	0.12	1	15624	9568
SD (dyad)	1.88	1.55	2.20	1	3449	5148
SD (mother)	1.66	0.78	2.66	1	1771	1305

Table S4. Posterior summaries for the home-range intersection-area model (hurdle Gamma).

Parameter	Median	HDI lo	HDI hi	Rhat	ESS bulk	ESS tail
Intercept (count)	-0.37	-0.68	-0.04	1.000	5307	6718
Intercept (shape)	1.05	0.88	1.21	1.000	7707	8263
Intercept (hurdle)	-5.83	-8.84	-3.14	1.000	5708	6706
β Offspring age difference	-0.06	-0.15	0.02	1.000	15358	9754
β Mean home-range area	0.44	0.36	0.52	1.000	15055	10353
β Offspring age (sum)	-0.04	-0.11	0.03	1.000	16392	9307
β Relatedness	0.46	0.28	0.64	1.001	5485	6455
β Fruit availability	-0.02	-0.10	0.06	1.000	11917	9372
β Vegetation greenness (EVI)	0.03	-0.05	0.10	1.000	13588	10097
β_{shape} Mean home-range area	0.58	0.41	0.76	1.000	13652	9838
β_{hu} Offspring age difference	0.33	-0.58	1.23	1.000	12309	9273
β_{hu} Mean home-range area	-2.24	-3.57	-1.12	1.000	8506	7908
β_{hu} Offspring age (sum)	0.27	-0.41	0.97	1.000	12994	8519
β_{hu} Relatedness	-2.95	-6.05	-0.35	1.001	6511	6791
β_{hu} Fruit availability	-0.10	-0.89	0.70	1.000	14457	9417
β_{hu} Vegetation greenness (EVI)	-0.64	-1.32	0.02	1.000	11575	8564
SD (dyad)	0.52	0.35	0.70	1.001	2104	4881
SD_{hu} (dyad)	2.95	1.57	4.56	1.000	4754	7167
SD (mother)	0.60	0.09	1.03	1.000	1791	1935
SD_{hu} (mother)	2.49	0.01	4.62	1.000	3047	3909
Cor (dyad, μ–hu)	-0.01	-0.50	0.48	1.000	2029	3686
Cor (mother, μ–hu)	-0.40	-0.94	0.19	1.000	4724	5860

Table S5. Posterior summaries for the infant–infant play-scan model (hurdle negative binomial). β rows refer to the count component; β_{hu} rows refer to the hurdle (zero) component.

Parameter	Median	HDI lo	HDI hi	Rhat	ESS bulk	ESS tail
Intercept (count)	-1.40	-3.38	0.49	1.000	4638	6233
Intercept (hurdle)	14.88	9.97	19.81	1.000	7055	7825
β Offspring age difference	-0.95	-1.41	-0.51	1.000	10234	8101
β Offspring age (sum)	1.37	0.90	1.82	1.000	8221	7155
β Offspring age (sum)^2	-1.06	-1.47	-0.65	1.001	8446	8126
β Relatedness	-0.11	-0.85	0.66	1.000	2231	2478
β Fruit availability	-0.30	-0.65	0.02	1.000	13046	9120
β Vegetation greenness (EVI)	-0.20	-0.52	0.15	1.000	12260	8651
β_{hu} Offspring age difference	0.47	0.12	0.83	1.000	12209	9343
β_{hu} Offspring age (sum)	-0.98	-1.33	-0.62	1.000	11357	9386
β_{hu} Offspring age (sum)^2	0.50	0.18	0.83	1.000	11659	8584
β_{hu} Relatedness	-1.42	-1.94	-0.94	1.000	7031	7526
β_{hu} Fruit availability	-0.55	-0.92	-0.20	1.000	10049	8770
β_{hu} Vegetation greenness (EVI)	0.21	-0.13	0.53	1.000	9879	9031
β_{hu} log(observation effort)	-1.19	-1.66	-0.69	1.000	10884	8293
SD (dyad)	0.35	0.00	1.59	1.000	1669	1911
SD_{hu} (dyad)	1.22	0.46	2.06	1.000	2416	2448
SD (mother)	3.31	0.72	6.10	1.000	1429	1738
SD_{hu} (mother)	2.20	0.01	4.21	1.001	1730	1754
Cor (dyad, μ–hu)	0.25	-0.50	0.91	1.000	1400	4066
Cor (mother, μ–hu)	-0.01	-0.67	0.65	1.000	3165	5965
Shape	0.61	0.35	0.86	1.000	7916	7717

Table S6. Posterior summaries for the daily path length model (three-level association/play encoding).

Parameter	Median	HDI lo	HDI hi	Rhat	ESS bulk	ESS tail
Intercept (count)	-0.31	-0.39	-0.23	1	2538	4598
β Association, no play	0.18	0.15	0.22	1	16289	9180
β Play day	0.34	0.28	0.41	1	16631	9153
β Offspring age	0.06	0.04	0.08	1	17004	9842
β Fruit availability	0.15	0.13	0.16	1	18880	9452
β Vegetation greenness (EVI)	0.03	0.02	0.05	1	19059	9621
SD (mother)	0.18	0.12	0.25	1	3240	5645
Shape	5.01	4.77	5.27	1	15679	9742

Table S7. Posterior summaries for the daily path length model (four-level adjacent-day encoding).

Parameter	Median	HDI lo	HDI hi	Rhat	ESS bulk	ESS tail
Intercept (count)	-0.33	-0.44	-0.23	1.001	2279	3784
β Day before play	0.09	-0.03	0.20	1.000	17280	9080
β Day of play	0.34	0.27	0.41	1.000	19668	8919
β Day after play	0.14	0.05	0.23	1.000	21804	8797
β Offspring age	0.05	0.02	0.07	1.000	16194	9447
β Fruit availability	0.16	0.14	0.19	1.000	15915	9143
β Vegetation greenness (EVI)	0.05	0.03	0.08	1.000	19496	8964
SD (mother)	0.21	0.13	0.30	1.000	3968	6946
Shape	5.02	4.66	5.35	1.000	15414	8902

Table S8. Posterior summaries for the feeding-proportion model (three-level association/play encoding).

Parameter	Median	HDI lo	HDI hi	Rhat	ESS bulk	ESS tail
Intercept (count)	0.16	0.05	0.28	1.000	1838	3060
β Association, no play	-0.15	-0.19	-0.11	1.000	10838	7393
β Play day	-0.25	-0.32	-0.18	1.000	11463	8948
β Offspring age	-0.02	-0.03	0.00	1.000	11616	9115
β Fruit availability	-0.07	-0.09	-0.05	1.000	9899	8324
β Vegetation greenness (EVI)	-0.02	-0.04	0.00	1.000	10847	7887
SD (mother)	0.24	0.16	0.33	1.001	2410	4556
ϕ	15.62	14.89	16.41	1.000	11125	8288

Table S9. Posterior summaries for the feeding-proportion model (four-level adjacent-day encoding).

Parameter	Median	HDI lo	HDI hi	Rhat	ESS bulk	ESS tail
Intercept (count)	0.12	-0.02	0.26	1.001	2092	3802
β Day before play	0.03	-0.08	0.16	1.000	12626	8515
β Day of play	-0.23	-0.30	-0.15	1.000	12153	8717
β Day after play	-0.04	-0.13	0.06	1.000	11592	8331
β Offspring age	0.02	-0.01	0.04	1.000	11079	8982
β Fruit availability	-0.06	-0.09	-0.04	1.000	12337	9095
β Vegetation greenness (EVI)	-0.03	-0.05	0.00	1.000	12166	8490
SD (mother)	0.31	0.20	0.43	1.000	2922	5102
ϕ	16.06	14.99	17.13	1.000	11780	8092

Table S10. Posterior summaries for the Approach model. β Day before play and Within-day before play are the categorical phase contrasts vs. baseline.

Parameter	Median	HDI lo	HDI hi	Rhat	ESS bulk	ESS tail
Intercept (count)	0.00	-0.02	0.01	1	9070	7303
β Day before play	0.09	0.02	0.16	1	18430	9141
β Within-day before play	-0.01	-0.11	0.09	1	11642	8724
β Relatedness	0.00	-0.01	0.01	1	12640	7835
β Mode separation	0.00	-0.01	0.01	1	13923	9284
β Offspring age difference	0.00	-0.01	0.01	1	18111	9260
β Offspring age (sum)	0.01	0.00	0.02	1	17903	9192
β Fruit availability	0.00	-0.02	0.01	1	15694	9615
β Vegetation greenness (EVI)	0.00	-0.01	0.01	1	18931	9234
β Daily path length	0.00	-0.01	0.01	1	17032	9229
β Window duration	-0.02	-0.04	0.00	1	12035	8416
SD (dyad)	0.01	0.00	0.02	1	4702	6068
SD (mother)	0.01	0.00	0.03	1	4676	5238
σ	0.28	0.27	0.29	1	20797	8477

Table S11. Posterior summaries for the Return model. β Within-day after play and Day after play are the categorical phase contrasts vs. baseline.

Parameter	Median	HDI lo	HDI hi	Rhat	ESS bulk	ESS tail
Intercept (count)	-0.02	-0.04	0.00	1.001	5522	6005
β Within-day after play	0.07	0.03	0.12	1.000	11836	9379
β Day after play	-0.01	-0.05	0.04	1.000	15857	9653
β Relatedness	0.00	-0.01	0.01	1.000	11797	9584
β Mode separation	0.00	-0.01	0.01	1.000	15041	9892
β Offspring age difference	0.00	-0.01	0.01	1.000	18257	9540
β Offspring age (sum)	0.00	-0.01	0.01	1.000	16640	8805
β Fruit availability	0.00	-0.01	0.01	1.000	14460	9644
β Vegetation greenness (EVI)	0.00	-0.01	0.01	1.000	18917	8481
β Daily path length	-0.01	-0.02	0.00	1.000	16468	9739
β Window duration	0.01	0.00	0.02	1.000	11475	9596
SD (dyad)	0.01	0.00	0.02	1.000	6338	5289
SD (mother)	0.02	0.01	0.04	1.001	3767	4340
σ	0.25	0.24	0.25	1.000	17534	9301

References

1. S Wright, Coefficients of inbreeding and relationship. *The Am. Nat.* **56**, 330–338 (1922).
2. MA van Noordwijk, et al., Reproductive success of Bornean orangutan males: scattered in time but clustered in space. *Behav. Ecol. Sociobiol.* **77**, 134 (2023).
3. MA Van Noordwijk, et al., Female philopatry and its social benefits among Bornean orangutans. *Behav. Ecol. Sociobiol.* **66**, 823–834 (2012).
4. N Arora, et al., Parentage-based pedigree reconstruction reveals female matrilineal clusters and male-biased dispersal in nongregarious asian great apes, the Bornean orang-utans (Pongo pygmaeus). *Mol. Ecol.* **21**, 3352–3362 (2012).
5. AM Ashbury, et al., Home range establishment and the mechanisms of philopatry among female Bornean orangutans (Pongo pygmaeus wurmbii) at tuanan. *Behav. Ecol. Sociobiol.* **74**, 42 (2020).
6. JM Calabrese, CH Fleming, E Gurarie, ctmm: an r package for analyzing animal relocation data as a continuous-time stochastic process. *Methods Ecol. Evol.* **7**, 1124–1132 (2016) _eprint: https://besjournals.onlinelibrary.wiley.com/doi/pdf/10.1111/2041-210X.12559.
7. CH Fleming, et al., Rigorous home range estimation with movement data: a new autocorrelated kernel density estimator. *Ecology* **96**, 1182–1188 (2015).
8. C Fleming, et al., Correcting for missing and irregular data in home-range estimation. *Ecol. Appl.* **28**, 1003–1010 (2018).

9. MJ Noonan, et al., Scale-insensitive estimation of speed and distance traveled from animal tracking data. *Mov. Ecol.* **7**, 35 (2019).
10. ER Vogel, et al., Nutritional ecology of wild Bornean orangutans (Pongo pygmaeus wurmbii) in a peat swamp habitat: Effects of age, sex, and season. *Am. J. Primatol.* **79**, 1–20 (2017).
11. SA Wich, et al., Forest fruit production is higher on Sumatra than on Borneo. *PLoS One* **6**, e21278 (2011).
12. A Huete, et al., Overview of the radiometric and biophysical performance of the modis vegetation indices. *Remote. sensing environment* **83**, 195–213 (2002).
13. SL Tuck, et al., Modistools–downloading and processing modis remotely sensed data in r. *Ecol. evolution* **4**, 4658–4668 (2014).
14. AJ Marshall, SA Wich, Characterization of primate environments through assessment of plant phenology in *Primate Ecology and Conservation: A Handbook of Techniques*, eds. EJ Sterling, N Bynum, ME Blair. (Oxford University Press, Oxford), pp. 103–127 (2013).
15. SR Jammalamadaka, A Sengupta, *Topics in circular statistics.* (world scientific) Vol. 5, (2001).
16. J Pearl, The causal foundations of structural equation modeling in *Handbook of Structural Equation Modeling*, ed. RH Hoyle. (Guilford Press, New York), pp. 68–91 (2012).
17. R McElreath, *Statistical rethinking: A Bayesian course with examples in R and Stan.* (Chapman and Hall/CRC), (2018).
18. ZM Laubach, EJ Murray, KL Hoke, RJ Safran, W Perng, A biologist's guide to model selection and causal inference. *Proc. Royal Soc. B: Biol. Sci.* **288** (2021).
19. J Textor, B Van der Zander, MS Gilthorpe, M Liśkiewicz, GT Ellison, Robust causal inference using directed acyclic graphs: the r package 'dagitty'. *Int. J. Epidemiol.* **45**, 1887–1894 (2016).
20. PC Bürkner, brms: An r package for bayesian multilevel models using stan. *J. statistical software* **80**, 1–28 (2017).
21. DA Kenny, L La Voie, The social relations model in *Advances in experimental social psychology.* (Elsevier) Vol. 18, pp. 141–182 (1984).
22. JM Koster, G Leckie, Food sharing networks in lowland nicaragua: an application of the social relations model to count data. *Soc. Networks* **38**, 100–110 (2014).
23. J Koster, G Leckie, A Miller, R Hames, Multilevel modeling analysis of dyadic network data with an application to ye'kwana food sharing. *Am. J. Phys. Anthropol.* **157**, 507–512 (2015).
24. K Kajokaite, A Whalen, J Koster, S Perry, Social integration predicts survival in female white-faced capuchin monkeys. *Behav. Ecol.* **33**, 807–815 (2022).
25. JB Silk, ER Roberts, BJ Barrett, SK Patterson, SC Strum, Female–male relationships influence the form of female–female relationships in olive baboons, Papio anubis. *Animal Behav.* **131**, 89–98 (2017).
26. OT Jacobson, MC Crofoot, GE Finerty, SE Perry, BJ Barrett, Environmental fluctuations alter the competitive trade-offs of group size in a social primate. *Nat. Ecol. & Evol.* **10**, 919–931 (2026).
27. WJ Browne, H Goldstein, J Rasbash, Multiple membership multiple classification (mmmc) models. *Stat. Model.* **1**, 103–124 (2001).
28. G Leckie, Multiple membership models in *Encyclopedia of quality of life and well-being research.* pp. 4533–4537 (2024).
29. J Rushmore, et al., Social network analysis of wild chimpanzees provides insights for predicting infectious disease risk. *J. Animal Ecol.* **82**, 976–986 (2013).
30. J Hart, MN Weiss, D Franks, L Brent, Bison: A bayesian framework for inference of social networks. *Methods Ecol. Evol.* **14**, 2411–2420 (2023).
31. JA Kunz, et al., Play behavior varies with age, sex, and socioecological context in wild, immature orangutans (Pongo spp.). *Int. J. Primatol.* **45**, 739–773 (2024).
32. MA van Noordwijk, et al., Development of independence: Sumatran and bornean orangutans compared in *Orangutans: Geographic Variation in Behavioral Ecology and Conservation*, eds. SA Wich, SS Utami Atmoko, TM Setia, CP van Schaik. (Oxford University Press), p. 189–203 (2008).
